# Novel Chitosan-Gelatin Scaffold with Valproic Acid Augments In Vitro Osteoblast Differentiation of Mesenchymal Stem Cells

**DOI:** 10.3390/jfb15090252

**Published:** 2024-08-31

**Authors:** Maha Alghofaily, Fahd Alsalleeh, Lamees Alssum, Manikandan Muthurangan, Musaad Alfayez, Michael D. Weir, Hockin H. K. Xu

**Affiliations:** 1 Restorative Dental Sciences, College of Dentistry, King Saud University, Riyadh 11541, Saudi Arabia; falsalleeh@ksu.edu.sa; 2 Department of Periodontics and Community Dentistry, College of Dentistry, King Saud University, Riyadh 11545, Saudi Arabia; lalssum@ksu.edu.sa; 3 Stem Cell Unit, Department of Anatomy, College of Medicine, King Saud University, Riyadh 11461, Saudi Arabia; mrangan@ksu.edu.sa (M.M.); alfayez@ksu.edu.sa (M.A.); 4 Department of Biomaterials and Regenerative Dental Medicine, University of Maryland School of Dentistry, Baltimore, MD 21201, USA; michael.weir@umaryland.edu (M.D.W.); hxu2@umaryland.edu (H.H.K.X.)

**Keywords:** chitosan, gelatin, scaffolds, histone deacetylase inhibitors, stem cells

## Abstract

The study aimed to develop a biodegradable scaffold incorporating valproic acid (VPA) for improved human bone marrow-derived mesenchymal stem cell (hBMSC) proliferation, differentiation, and bone mineral synthesis. A chitosan–gelatin (CH-G) scaffold was fabricated and loaded with varying concentrations of VPA (1, 3, 5 mM/L). In vitro studies assessed drug release, cell proliferation, morphology, mineralization, and gene expression. VPA was rapidly released from the scaffold, with over 90% cumulative release within seven days. Cells cultured on VPA-loaded scaffolds exhibited significantly enhanced proliferation and mineralization compared to the control. VPA treatment upregulated osteocalcin and runt-related transcription factor 2 (Runx-2) expression, key markers of osteogenic differentiation. The CH-G scaffold, particularly with 1 mM/L VPA, demonstrates excellent biocompatibility and promotes hBMSC-mediated bone regeneration. This novel approach holds promise for future applications in bone tissue engineering.

## 1. Introduction

Novel dental materials that specifically target genetic and epigenetic pathways could potentially make significant contributions to the reparative processes involved in tissue regeneration, as demonstrated by the advancements in gene detection approaches [1,2]. Epigenetic modulators regulate the expression of genes in the genome without altering the base sequence of DNA [3], thereby controlling the cell phenotype and regulating the renewal of stem cell populations [4,5]. The nuclear enzyme activities of histone deacetylase inhibitors (HDIs), a frequently studied topic, are recognized as potential targets for pharmacologically enhancing stem cell differentiation and altering the fate of the cell [6]. Types of HDIs, including valproic acid (VPA), trichostatin A, and butyric acid, have been studied within the realm of regeneration research. These compounds can potentially induce cell proliferation and differentiation, in addition to having anti-inflammatory effects [7,8]. VPA is widely used in the treatment of various neurological disorders [9,10].

Epigenetic mechanisms play a pivotal role in regulating stem cell fate and differentiation [1]. Recent studies have highlighted the importance of DNA methylation and histone modifications in governing the multipotency of mesenchymal stem cells (MSCs) [3]. Previous research has reported VPA-mediated enhancement of proliferation in hematopoietic stem cells and neuronal cells [11,12]. Moreover, studies unveiled the osteoinductive potential of VPA in murine and human MSCs, characterized mainly by upregulated expression of runt-related transcription factor 2 (Runx-2), the master osteogenic transcription factor, and other osteoblast-specific markers [13,14]. Therefore, VPA could be useful for in vivo bone engineering using human MSCs. 

Regenerative medicine demands biomaterials capable of controlled drug delivery to optimize tissue repair. Chitosan (CH), a natural polymer, has emerged as a promising scaffold material due to its biocompatibility, antimicrobial properties, and ability to promote cell adhesion and proliferation [15,16,17]. While CH-based scaffolds have shown potential for various tissue engineering applications, their efficacy in delivering therapeutic agents like VPA for stem cell modulation remains largely unexplored. Therefore, the study aimed to investigate the potential of VPA-loaded chitosan scaffolds to influence hBMSC behavior and induce tissue mineralization. The hypothesis was that VPA-loaded chitosan-gelatin scaffolds would significantly enhance hBMSC proliferation and osteogenic differentiation compared to control scaffolds lacking VPA. Thus, the objective of this study was two-fold: (1) to develop a novel CH-gelatin scaffold that can release VPA and (2) to evaluate the proliferation and osteogenic differentiation of hBMSCs in the presence of VPA-releasing CH-gelatin scaffold.

## 2. Materials and Methods

### 2.1. Ethical Approval

The Institutional Review Board (IRB) approved all the study experiments following relevant rules and regulations (Project No. E-21-6214). 

### 2.2. Fabrication of Chitosan-Gelatin Scaffolds

A chitosan–gelatin (CH-G) scaffold was used to deliver various concentrations of VPA. The scaffold was constructed as described previously [17,18]. A 2% solution of CH (75–85% deacetylated, mol wt 310,000–375,000; Sigma-Aldrich; St. Louis, MO, USA) was dissolved in 1% (*v*/*v*) aqueous solution of glacial acetic acid (Sigma-Aldrich; Darmstadt, Germany) and added to 3% gelatin (Sigma-Aldrich; St. Louis, MO, USA) dissolved in water and mixed at 37 °C. The homogenous mixture was poured into a customized mold (area: 5 mm^2^; thickness: 3 mm^2^), refrigerated at 4 °C, and frozen at −80 °C for 4 h. The mixture was then lyophilized to acquire the three-dimensional (3D) scaffold.

The samples were neutralized and sterilized using Gamma radiation at 25 kGy.

### 2.3. Preparation of VPA

The concentrations of VPA were selected based on the optimum concentrations without exerting a cytotoxic effect [8]. One M stock solution of VPA (2-propylpentanoic acid sodium salt) (Sigma-Aldrich) in PBS (Sigma-Aldrich) was diluted to a range of experimental concentrations (1, 3, and 5 mM/L) [19]. The resulting groups are referred to as:

Group 1: VPA-1; Group 2: VPA-3; Group 3: VPA-5; and control: scaffold.

### 2.4. VPA Loading and Incubation

The dry CH-G scaffold samples were placed in 24-well plates and immersed in 1 mL of VPA (1, 3, or 5 mM/L), and the control samples were immersed in phosphate-buffered saline (PBS) (pH 7.4). All samples were left overnight in an incubator at 37 °C. After incubation, the hydrated scaffolds were cautiously transferred to new PBS in 24-well plates and kept in an incubator at 37 °C until further investigation.

### 2.5. Determination of VPA Releases from CH-G Scaffold

A spectrophotometer determined in vitro drug release from the scaffold in 5 mL of phosphate buffer (pH 7.2). A calibration curve was generated using VPA-associated absorbance at 400 nm and measured using a UV-Vis spectrophotometer (Shimadzu, Kyoto, Japan, 1900). The UV absorbance of all groups was measured by withdrawing a 2 mL aliquot from each sample and replenishing it with equal amounts of fresh PBS. The measurements were acquired following time intervals: 1 h, 2 h, 4 h, 6 h, 8 h, and 24 h. Subsequently, measurements were acquired once a day for 7 days. The release percentage of the corresponding medicaments was calculated from the absorbance value using antibiotics as per the following equation:

$$=\frac{\sum_{t\to 0}^{t}M_{t}}{M_{\begin{array}{c} Actual \end{array}}}\times 100\%$$

The variable “*M*” represents the amount of VPA released at a specific point during the measurement period. *M_t_* denotes the initial amount of the drug present in the scaffold material. The symbol *M_Actual_* represents the release exponent, a mathematical depiction of the drug’s release pattern over time.

### 2.6. Cell Culture and Seeding

Immortalized human bone marrow mesenchymal stem cells (hTERT-MSC) were a generous gift from Professor Moustapha Kassem, University Hospital of Odense, Denmark. Cell culture was performed as previously described [20,21]. Briefly, hTERT-MSCs were maintained in Dulbecco’s Modified Eagle’s Medium (DMEM; Gibco-Invitrogen, Waltham, MA, USA) supplemented with 10% fetal bovine serum (FBS), 110 mg/L sodium pyruvate, 4500 mg/L D-glucose, 4 mM L-glutamine, 100 U/mL penicillin–streptomycin, and non-essential amino acids. Cells were incubated at 37 °C in a humidified atmosphere containing 5% CO_2_. For each experiment, cells were cultured for 24 h before adding CH-G scaffolds under various experimental conditions, including a control group. All conditions were tested in triplicate with three wells per condition.

### 2.7. Cell Viability and Proliferation

Cell proliferation was assessed using the AlamarBlue assay (Thermo Fisher Scientific, Waltham, MA, USA) according to the manufacturer’s protocol. Briefly, 1 × 10^6^ cells were seeded per well in a 24-well plate and allowed to adhere. Cells were then covered with experimental scaffold groups or control and cultured for three days. Cell metabolic activity, indicative of cell proliferation, was determined by measuring fluorescence intensity (Ex/Em: 530 nm/590 nm) using a fluorescence reader (BioTek^®^, Winooski, VT, USA).

### 2.8. Live/Dead Staining

A dual fluorescent staining solution (1 µL) comprising 100 µg/mL of acridine orange and 100 µg/mL of ethidium bromide (AO/EB, Sigma, St. Louis, MO, USA) was used to differentiate between living and dead cells. Cells exposed to scaffolds with and without VPA were stained with the solution. The cells were analyzed, documented, and quantified using a Nikon Eclipse Ti fluorescence microscope (Nikon, Tokyo, Japan).

### 2.9. Characterization of hBMSCs in CH-G Scaffolds by Scanning Electron Microscopy 

A Scanning Electron Microscope (SEM) (JSM-6360LV SEM; Jeol, Tokyo, Japan) was used to assess the morphological alterations in the scaffold cells. The SEM analysis of the materials was followed by photography. The scaffolds with or without VPA were then placed over the cells and incubated for three days. For each sample, digital pictures were acquired at a 1000× magnification.

### 2.10. Alkaline Phosphatase (ALP) Quantification Assay and Staining

Using a quantification kit and a stating kit (BioVision ALP activity colorimetric assay; Bio Vision, Inc., Milpitas, CA, USA) following the manufacturer’s instructions, the metabolic activity at 9 and 14 days was evaluated and absorbance was read at 405 nM using fluorescence reader (BioTek^®^, Winooski, VT, USA).

Furthermore, the ALP staining was assessed on day 9 of hBMSC differentiation. Cells were washed with phosphate-buffered saline and fixed in acetone/citrate buffer for 5 min at room temperature. Following fixation, cells were incubated with Naphthol AS-TR phosphate substrate and Fast Red solutions (0.417 mg/mL) (Sigma-Aldrich (St. Louis, MO, USA)), for 1 h at room temperature. After thorough washing with water, ALP-positive cells were visualized and imaged under a microscope. 

### 2.11. Alizarin Red-S Staining Assay

The ability of hBMSCs to form calcified nodules was evaluated on day 12 using the Alizarin Red-S Staining Assay, either with or without VPA. Then, each well was treated with 2% ARS solution (ScienCell, Carlsbad, CA, USA) according to manufacturer instructions. Images were taken with a Nikon Eclipse fluorescent microscope (Nikon, Tokyo, Japan) after the cells had been washed with water.

### 2.12. Quantitative Real-Time Polymerase Chain Reaction (qRT-PCR) for Gene Analysis

On days 9 and 12, total RNA was extracted using an RNA Mini Kit (Analytik Jena AG; Jena, Germany, REF No:845-KS-2040250) as per the manufacturer’s guidelines. The steps were followed as described previously [22]. The expression levels of the reference genes, osteonectin, runt-related transcription factor 2 (Runx-2), osteocalcin, collagen Type 1, and alkaline phosphate (ALP), were evaluated. To compute the relative gene expression using a comparative CT approach, the data were normalized to the reference gene, glyceraldehyde-3-phosphate dehydrogenase (GAPDH), where Delta-delta CT (CT) is the difference between the CT values of the target and reference genes. Table 1 contains a list of the primer sequences used in this investigation.

### 2.13. Statistical Analysis

The statistical analyses utilized SPSS software (ver. 24.0; IBM Corp., Armonk, NY, USA). The data obtained from each trial were examined using one-way ANOVA. Tukey’s post hoc test was employed to compare the variations between groups at the same time point. The significance threshold was chosen at *p* = 0.05.

## 3. Results

### 3.1. CH-G Scaffold Porosity and Morphology

**Figure 1:**
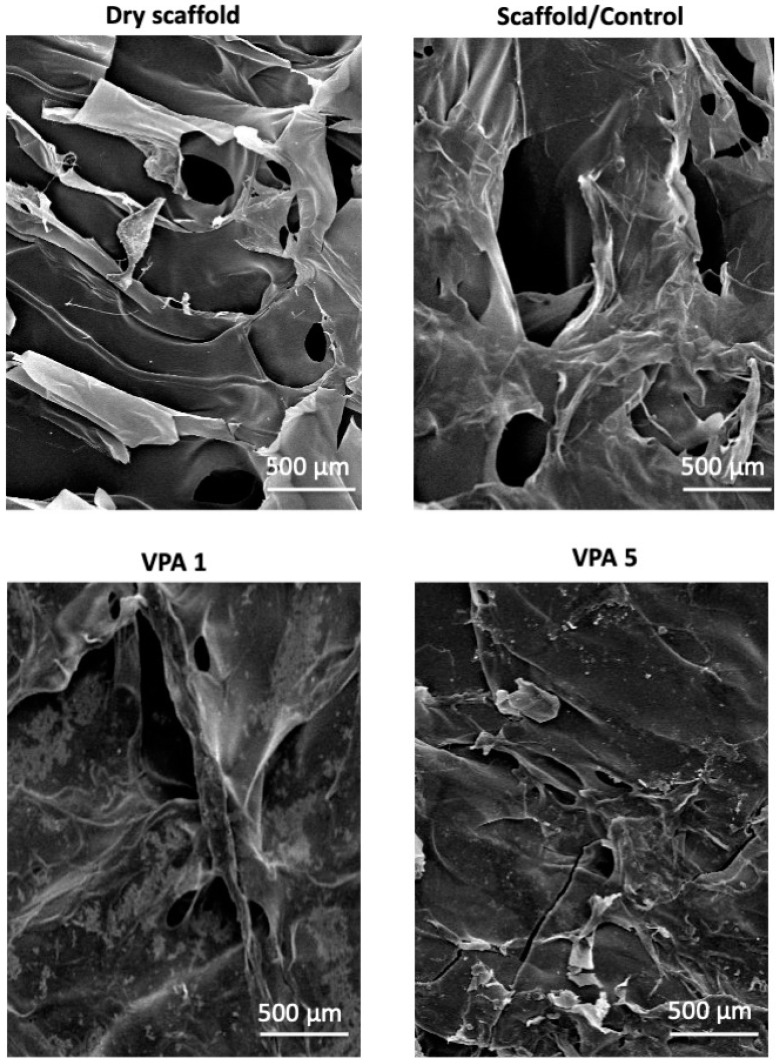
SEM images of CH-G scaffolds. Representative SEM micrographs of dry and hydrated CH-G scaffolds treated with different VPA concentrations (1 and 5 mM). Scale bar = 500 μm. The dry scaffold exhibits a highly porous, interconnected structure. Hydrated scaffolds demonstrate a reduction in pore size following VPA treatment.

The scaffold exhibited a highly porous, interconnected structure with a mean pore diameter of 56 µm and a 31–69 µm range, as observed by SEM at 5000× magnification (Figure 1). Notably, infusion with VPA resulted in a discernible increase in scaffold size and pore formation.

### 3.2. Differential Release of VPA from the CH-G Scaffold

**Figure 2:**
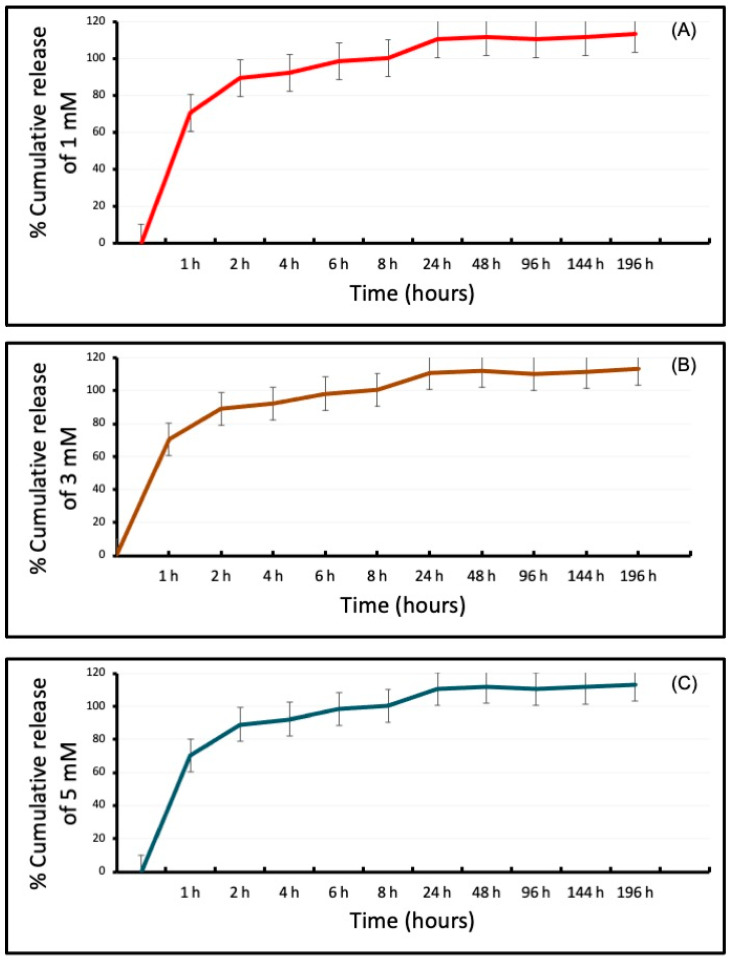
Invitro Cumulative release (%) profile of VPA at (**A**) 1 mM, (**B**) 3 mM, and (**C**) 5 mM from CH-G scaffolds (*n* = 4 in triplicate).

The initial burst release of the drug from the scaffold had a similar release rate among the experimental groups during the first hour. All groups reached a plateau before 24 h, and more than 90% of the cumulative release was observed for up to seven days (Figure 2).

### 3.3. VPA Enhances Cell Proliferation and Improves Viability 

**Figure 3:**
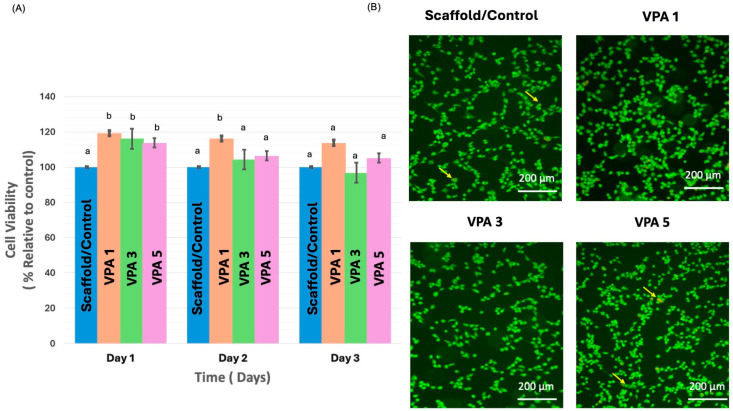
Cell viability and morphology on CH-G scaffolds. (**A**) Cell proliferation of hBMSCs exposed to different experimental groups compared to control, as assessed by AlamarBlue assay. Different lowercase letters (a, b) indicate statistical significance compared to control. Data represent mean ± standard deviation. (**B**) Representative fluorescent images of hBMSCs cultured on CH-G scaffolds with various VPA concentrations and control for three days. Cell viability was assessed using calcein-AM/PI staining (green: live cells; red: dead cells). Scale bar = 200 µm.

AlamarBlue data were collected from the experimental (VPA 1, VPA 3, and VPA 5) and CH-G scaffold control groups on days 1, 2, and 3. The results are shown in (Figure 3A). Regarding cell viability, the values observed in the VPA 1 group were significantly higher than those observed in the scaffold/control group on days 1 and 2 (*p* = 0.007 and *p* = 0.020, respectively). Similarly, the values observed in the VPA 3 and VPA 5 groups were significantly higher than those observed in the scaffold/control group on day 1 (*p* = 0.024 and *p* = 0.002, respectively). On day 3, all groups showed similar cell viability to the control group. 

Live–dead staining revealed no cytotoxicity in the experimental groups, and their results were comparable to those of the control group (Figure 3B).

### 3.4. Implications of VPA on Cell Morphology

**Figure 4:**
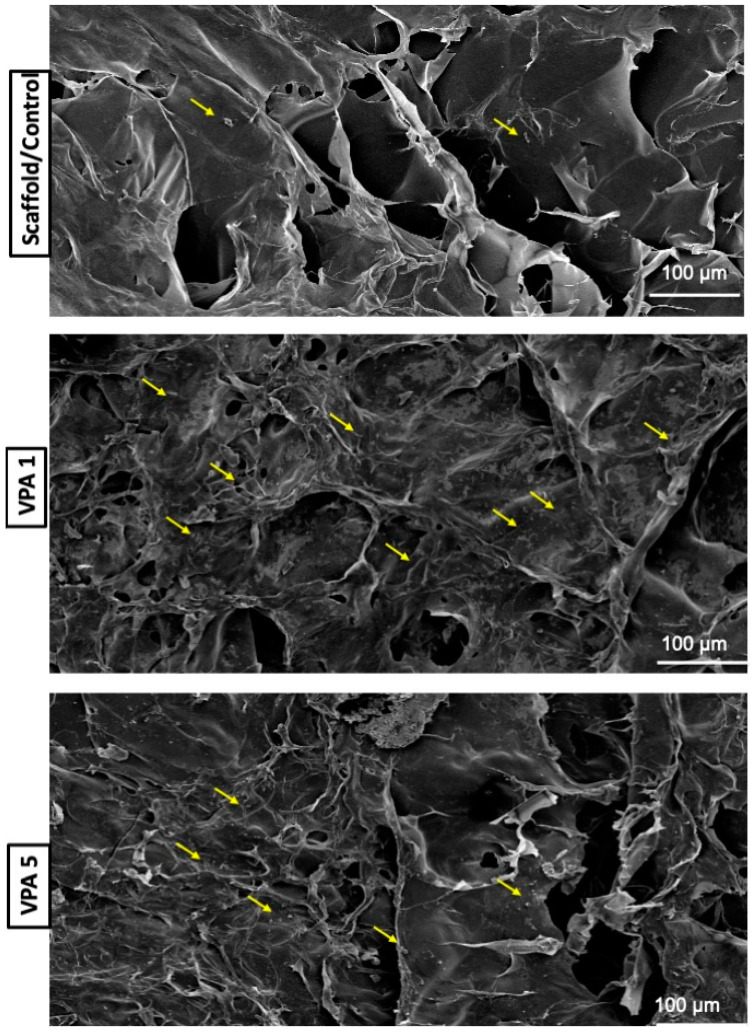
SEM image analysis of hBMSCs after three days of exposure to the experimental and control groups. Yellow arrows indicate cells (scale bar = 100 µm).

SEM examination of the cells on the scaffold with and without various concentrations of VPA performed three days later revealed different cell morphologies. The number of cells in the control group (without VPA) was lesser, and the cells appeared unattached to the cytoplasmic extensions. The VPA 1 group had abundant cells with rounded edges attached to the scaffold. In contrast, hBMSCs in the VPA 5 group were fewer and appeared detached from the cytoplasmic extension (Figure 4).

### 3.5. VPA Enhances Osteoblast Differentiation 

**Figure 5:**
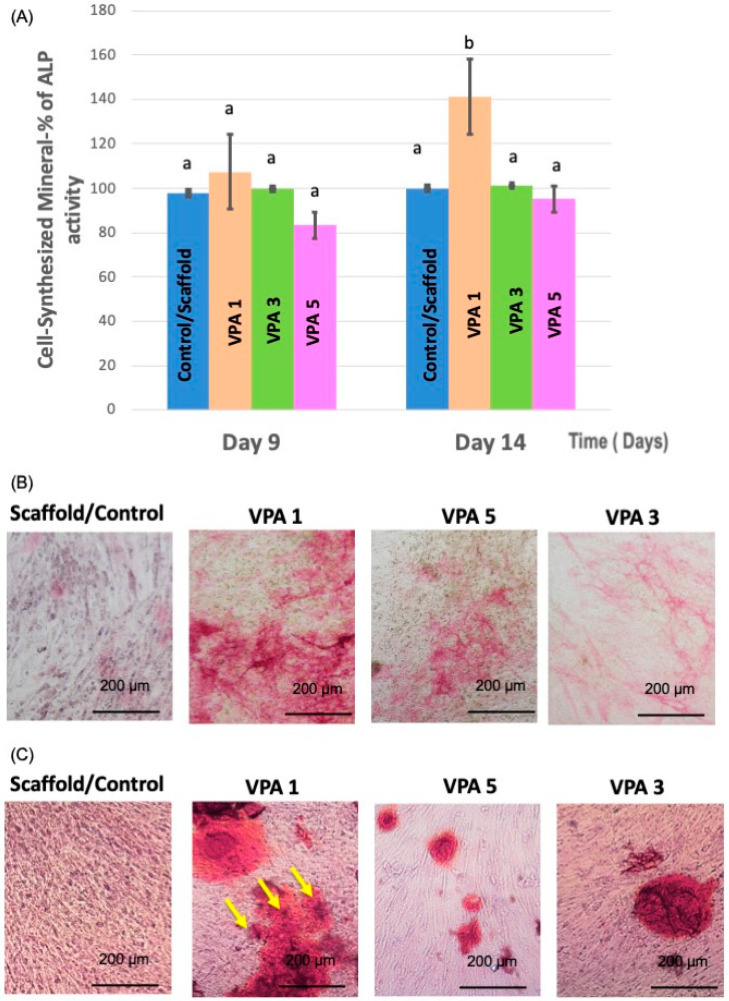
(**A**) Comparison of ALP activity of hBMSCs cells exposed to experimental groups and the control. Data represent mean ± SD. Different lowercase letters (a, b) indicate statistical significance compared to control. (**B**) Illustrations of the ALP enzyme in action at day 9. (**C**) Alizarin Red-S (ARS) staining at day 12. With VPA 1, observe a greater number of calcified nodule formations, as shown with yellow arrows (scale bar = 200 µm).

Quantitative and qualitative measurements were obtained using ALP assay at two time points on days 9 and 14. The quantitative assay revealed that compared with the control and other experimental groups, mineralization increased significantly following treatment with 1 mM/L VPA only on day 14 (*p* < 0.05). No significant increase in mineralization was observed following treatment with 3 or 5 mM/L of VPA at different time points when compared with the control (Figure 5A).

ALP staining was performed to confirm osteogenic differentiation in the qualitative assay. Osteogenic differentiation was more intense in the VPA 1 group (Figure 5B).

On day 12, the calcium nodule density was higher in all experimental groups compared to the control group. In comparison to the control and experimental groups, ARS staining was considerably higher in the VPA 1 group (Figure 5C).

### 3.6. VPA Modulates Osteogenic Gene Expression in hBMSCs

**Figure 6:**
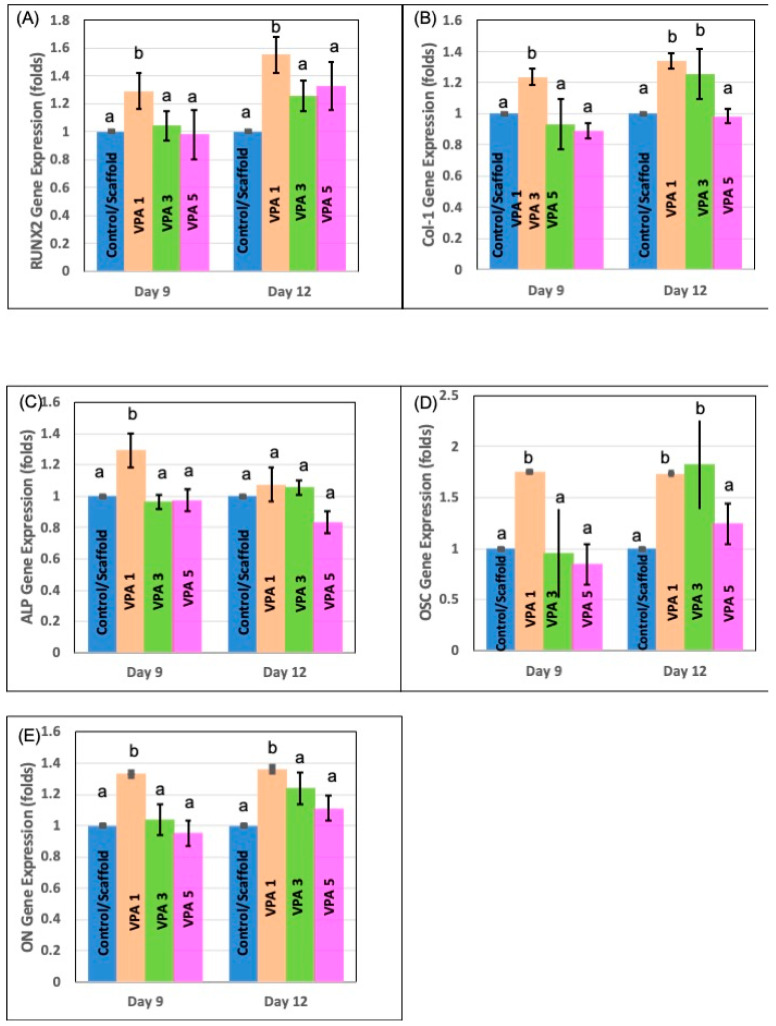
Results from qRT-PCR performed on days 9 and 12 following osteoblast induction. In (**A**) RUNX-2, (**B**) COL-1, (**C**) ALP, (**D**) osteocalcin, and (**E**) ON gene expression levels were examined and normalized to GAPDH. Data are presented as mean ± SD (*n* = 4 in triplicate). Different lower cases indicate statistical significance when compared with the control. (*p* < 0.05) at each time point. (Control: cells in Scaffold, [Runx-2: Runt-related transcription factor, Col-1: Collegen-1, ALP: Alkaline Phosphatase, OSC: Osteocalcin, ON: Osteonectin]).

On day 12, the VPA1 group exhibited a significant upregulation of key osteogenic genes, with osteocalcin and Runx-2 expression increased by 1.8-fold and 1.6-fold, respectively, compared to the control. The VPA3 group also demonstrated upregulated osteocalcin and collagen type 1 expression on day 12, mirroring the VPA1 group. Interestingly, a marked downregulation of ALP expression was observed in the VPA1 group on day 12, while ALP levels in other experimental groups remained comparable to the control on day 9 (Figure 6).

## 4. Discussion

This investigation represents a pioneering effort to explore the influence of VPA, delivered via a three-dimensional CH-G scaffold, on hBMSCs viability and osteogenic differentiation. Our findings underscore the potential of this novel delivery system to induce reparative processes and promote cellular differentiation within a scaffold-mediated environment. The microstructural characteristics of the CH-G scaffold, as revealed by SEM analysis, exhibited pore dimensions comparable to those reported in a previous study [17]. This pore architecture facilitates cell attachment, proliferation, and subsequent tissue formation [18,23]. These results suggest that the CH-G scaffold provides a suitable microenvironment for cellular interactions and potential tissue regeneration. 

The integration of gelatin within the CH scaffold was instrumental in defining its biological properties. Previous studies on nerve cells demonstrated that gelatin, when combined with chitosan, enhances cell adhesion and mineralization [24], prompting its inclusion in our formulation. While chitosan alone exhibited biocompatibility and minimal immune response, it limited cell spreading [25]. The VPA-loaded CH-G scaffold demonstrated sustained drug release throughout the experimental period, initiated by a rapid release phase. This release profile is characteristic of hydrophilic matrices [26]. The scaffold’s interconnected porous structure facilitated efficient water absorption, contributing to the observed drug release kinetics. 

Immortalized hBMSCs were employed in this study. These cells were generated by overexpressing the human telomerase reverse transcriptase gene in primary hBMSCs. Extensive characterization has confirmed that these immortalized hBMSCs retain the essential phenotypic and molecular characteristics of their primary counterparts, as reported by other studies [27,28]. This cell line model offers a consistent and reliable system for investigating the cellular responses to experimental conditions, circumventing the limitations associated with primary cell cultures.

The current investigation outlined a significant enhancement in cell proliferation and attachment on scaffolds treated with 1 mM/L VPA compared to untreated controls. Notably, cell viability remained unaffected across all VPA concentrations, and a marked increase in cell proliferation was observed within the first two days of 1 mM/L VPA exposure. These findings align with previous reports highlighting the beneficial effects of low-dose VPA on primary dental pulp, hematopoietic stem, and neuronal cells [11,12]. However, the literature also presents conflicting data regarding VPA’s impact on cellular behavior. Studies involving MSCs derived from various tissues have reported inhibited proliferation and self-renewal at VPA concentrations ranging from 0.4 to 10 mM [14,29,30]. In contrast, our data revealed a significant increase in cell viability during the initial two days of 1 mM/L VPA treatment without inducing cytotoxicity at higher concentrations. Furthermore, prolonged VPA exposure did not stimulate sustained cell proliferation. These observations suggest that the optimal VPA concentration for promoting cell proliferation is cell type-specific and may be influenced by factors such as treatment duration and delivery method.

The present study demonstrated that VPA loaded in CH-G scaffold significantly enhanced osteogenic differentiation of immortalized hBMSCs. This effect was evident at the lowest concentration of 1 mM/L after 9 and 14 days of treatment, as indicated by increased ALP activity. On day 12, to coincide with the expected period of active mineralization, calcium nodule formation was confirmed. Furthermore, gene expression analysis revealed a substantial upregulation of osteocalcin and Runx-2 by 1.8- and 1.6-fold, respectively. These findings align with previous reports demonstrating the osteogenic potential of HDIs, including VPA, in primary osteoblasts and various MSC lines [14,29,31,32,33,34]. Notably, VPA has been shown to preferentially induce osteogenic differentiation in MSCs [35]. A more recent study in mouse MSCs implicated microRNA-21 and the mitogen-activated protein kinase/extracellular signal-regulated kinase signaling pathway in VPA-mediated osteoblast differentiation and mineralization, highlighting the potential involvement of Runx-2 [13]. Future investigations should focus on delineating the upstream and downstream signaling pathways involved in this process to elucidate the underlying mechanisms of VPA loaded in CH-G-induced osteogenesis in our immortalized hBMSC model. A question arises about the superior mineralization observed with VPA 1 mM, and we acknowledge that this is an intriguing finding. While further investigation is required to fully elucidate the underlying mechanisms, we hypothesize that the optimal concentration of VPA may be crucial in balancing osteogenic differentiation and mineralization. It is possible that VPA 1 mM strikes a balance between stimulating osteogenic gene expression and promoting mineral deposition.

While the current study provides valuable insights into VPA-loaded CH-G’s proliferation and osteogenic potential, several limitations warrant consideration. The observed effects on proliferation and osteogenic differentiation suggest a complex interplay of factors requiring further investigation. Additionally, the in vitro nature of this study and the use of cell lines restrict the translational potential of the findings. The application of VPA as a single administration, rather than cyclic, limits our understanding of the optimal dosing regimen and potential long-term effects. To fully elucidate these findings’ underlying mechanisms and clinical relevance, future studies should explore the effects of VPA-loaded CH-G in animal models and various cell types. 

## 5. Conclusions

This study successfully developed a novel biodegradable CH-G scaffold incorporating VPA for potential bone tissue engineering applications. The results demonstrate that the controlled release of VPA from the CH-G scaffold significantly enhanced hBMSCs proliferation and osteogenic differentiation compared to the control group. The increased ALP activity, calcium nodule formation, and upregulation of osteogenic markers, osteocalcin, and Runx-2 underscore the potential of this VPA-loaded scaffold to promote bone mineralization. These findings suggest that the CH-G scaffold with 1 mM/L VPA holds promise as a promising biomaterial for bone regeneration. Further, in vivo studies are warranted to evaluate its efficacy in promoting bone repair and regeneration in animal models.

## Figures and Tables

**Table 1:** The primer sequences used in this study.

Primer	Forward	Reverse
**Alkaline phosphate**	5′-GGAACTCCTGACCCTTGACC-3′	5′-TCCTGTTCAGCTCGTACTGC-3′
**Osteocalcin**	5′-GGCAGCGAGGTAGTGAAGAG-3′	5′-CTCACACACCTCCCTCCTG-3′
**Runx-2**	5′-GTA GAT GGA CCT CGG GAA CC3′	5′-GAG GCG GTC AGA GAA CAA AC-3′
**Osteonectin**	5′-GAGGAAACCGAAGAGGAGG-3′	5-GGGGTGTTGTTCTCATCCAG-3′
**Collagen Type 1**	5′-GAGTGCTGTCCCGTCTGC-3′	5-TTTCTTGGTCGGTGGGTG-3′
**GAPDH**	5′-GAAGGTGAAGGTCGGAGT-3′	5′-GAAGATGGTGATGGGATTTC-3′

## Data Availability

The data of this study are available from the corresponding authors upon reasonable request.
